# Virologists—Heroes need weapons

**DOI:** 10.1371/journal.ppat.1006771

**Published:** 2018-02-08

**Authors:** Franziska Hufsky, Bashar Ibrahim, Martin Beer, Li Deng, Philippe Le Mercier, Dino P. McMahon, Massimo Palmarini, Volker Thiel, Manja Marz

**Affiliations:** 1 European Virus Bioinformatics Center, Jena, Germany; 2 RNA Bioinformatics and High-Throughput Analysis Jena, Friedrich Schiller University Jena, Jena, Germany; 3 Institute of Diagnostic Virology, Friedrich-Loeffler-Institute, Greifswald, Germany; 4 Institute of Virology, Helmholtz Zentrum Munich, Munich, Germany; 5 Swiss-Prot group, SIB, CMU, University of Geneva Medical School, Geneva, Switzerland; 6 Host parasite evolution and ecology, Institute of Biology, Free University of Berlin, Berlin, Germany; 7 Department for Materials and Environment, BAM, Federal Institute for Materials Research and Testing, Berlin, Germany; 8 MRC-University of Glasgow Centre for Virus Research, Glasgow, United Kingdom; 9 Federal Department of Home Affairs, Institute of Virology and Immunology, Bern and Mittelhäusern, Switzerland; 10 Department of Infectious Diseases and Pathobiology, University of Bern, Bern, Switzerland; The Fox Chase Cancer Center, UNITED STATES

Virologists. You might know a couple of them, but unless you are a virologist yourself, the probability that you have collaborated with one in the past is low. The community is relatively small, but they pack a heavy punch and are expected to play a leading role in the research into pathogens that lies ahead. You may ask why we think virologists are our future. Suffice it to say that it is not just because they have invented technologies that belong to the space age, including use of viruses as vehicles to shuttle genes into cells[1], organic nanoparticles with specific tools attached to their surfaces to get inside target cells[2], and using genetically modified viruses as therapies to fight against cancer[3]. Did you know that virologists currently only know of about 3,200 viral species but that more than 320,000 mammal-associated viruses[4] are thought to await discovery? Just think about the viruses hidden in the Arctic ice[5] or in the insects and other animals from once cut-off regions in the world, which now face ever-increasing human exposure[6]. But a heroic (as well as an apocalyptic) role for virologists may also be on the horizon, as the adoption of phage therapy may, in the future, be used to control harmful bacteria when antibiotics fail[7].

Bioinformaticians. You may know a couple of them, but usually you “just” need one to do proper math for your -omics experiments. Nowadays, nearly everyone in the life sciences has used BLAST [8] at least once, or made an alignment, or asked a bioinformatician to analyze high-throughput sequencing data. Of course, bioinformaticians do more than straightforward tasks. Software is based on algorithms and on data structures. Biological data are intrinsically complicated, and it is demanding to find appropriate data structures to process them efficiently[9]. Bioinformaticians routinely have to develop tailored, study-specific algorithms and tools used by a wide variety of scientists, including biochemists, biologists, geneticists, and molecular life scientists; but we rarely find virus-specific tools used by virologists. Why is this?

It is possibly not the problem of virologists but of bioinformaticians themselves. Although it was a virus that was the first organism to have its genome completely sequenced[10], bioinformaticians quickly focused attention on larger organisms, including humans, mice, plants, fungi, and bacteria. Perhaps there was no time left to take care of viruses? It also may be that, to a bioinformatician, a virus may appear uninteresting, at least at first. For example, the most dangerous human pathogenic (mostly RNA) viruses are short, usually contain only a single-digit number of genes that lack introns, contain only mononucleotide or dinucleotide repeats, and very few regulatory elements[11]. But astonishingly, we now know that the human genome consists of 8%–60% virus-derived sequences (depending on how this is measured: 8% can be directly traced back to viruses, whereas a figure of 60% includes LINEs and SINEs that are thought to be of viral origin[12]). Viruses have therefore played a very large part in shaping the evolution of the human genome as well as the genomes of organisms from all domains of life. Furthermore, we can observe evolution in viruses in realtime thanks to their extraordinary mutation rates, rapid replication cycles, large population sizes, and immense recombination potential[13]; this means that we are able to observe molecular evolution in a matter of days! Can you imagine that nearly all existing bioinformatical tools are not specifically designed for the context of viruses? We argue that without bioinformaticians, virologists are fighting with one arm tied behind their backs.

Clearly virologists are well positioned to tackle some of the major disease threats that will inevitably face humans in the decades to come—but they are currently not the best equipped. In order to give virologists weapons, i.e., bioinformatic tools, we recently founded the European Virus Bioinformatics Center (EVBC).

As alluded to above, this Center was developed based on our belief that we lack bioinformatical tools specifically designed for virology. We urgently need to develop algorithms for multiple genome alignments. It sounds trivial, but we still have no tool that can align, for example, 500,000 full-length coronaviruses (approximately 32,000 nt). We cannot visualize such alignments that are clearly needed to rapidly identify mutational hotspots and compensatory mutations.

Can you believe that there is not even a unified database for viruses? We only have good databases for Influenza (EpiFlu [14]), HIV [15], and human pathogenic viruses (ViPR [16]). There is the possibility to archive viral sequences in NCBI, but virologists use this facility only rarely because of its limited utility. For instance, submitters are asked to commit the name of a chromosome, which clearly is not applicable to viruses.

These examples emphasize that we need to bring virologists and bioinformaticians together. Again, this seems obvious and, perhaps, trivial. But in reality, they speak different languages. We urgently need young scientists that understand both virology and bioinformatics so that they can bridge the gap between these two complementary yet different disciplines.

The EVBC aims to develop bioinformatical tools for nearly all areas: (1) for detection of viruses, e.g., from high-throughput sequencing data; (2) virus assembly; (3) quasispecies reconstruction; (4) intraviral interactions; (5) virus entry, i.e., protein—protein interaction; (6) virus—host interactions; (7) phylogeny/cophylogeny; and (8) therapy. For the latter two areas, we have already established tools for HIV and Influenza A, but a large number of outstanding research questions remain. Finally, the EVBC will initiate and coordinate ring trials, undergraduate courses, graduate summer schools, and courses for principal investigators.

Interested? Take a look here: evbc.uni-jena.de.
